# Human iPSC-Derived Retinal Pigment Epithelium: A Model System for Prioritizing and Functionally Characterizing Causal Variants at AMD Risk Loci

**DOI:** 10.1016/j.stemcr.2019.04.012

**Published:** 2019-05-09

**Authors:** Erin N. Smith, Agnieszka D'Antonio-Chronowska, William W. Greenwald, Victor Borja, Lana R. Aguiar, Robert Pogue, Hiroko Matsui, Paola Benaglio, Shyamanga Borooah, Matteo D'Antonio, Radha Ayyagari, Kelly A. Frazer

**Affiliations:** 1 Department of Pediatrics, University of California San Diego, La Jolla, CA 92093, USA; 2 Institute for Genomic Medicine, University of California San Diego, 9500 Gilman Drive, La Jolla, CA 92093, USA; 3 Bioinformatics and Systems Biology Graduate Program, University of California, San Diego, La Jolla, CA 92093, USA; 4 Programa de Pós-Graduação em Ciências Genômicas e Biotecnologia, Universidade Católica de Brasília, Brasília, DF, Brazil; 5 Centre for Clinical Brain Sciences, School of Clinical Sciences, The University of Edinburgh, Edinburgh, UK; 6 Shiley Eye Institute, University of California San Diego, La Jolla, CA 92093, USA; 7 Cellular and Molecular Medicine East, 9500 Gilman Drive #0761, La Jolla, CA 92093-0761, USA

**Keywords:** induced pluripotent stem cells, retinal pigment epithelium, age-related macular degeneration, VEGFA, chromatin accessibility, fine mapping, regulatory variants, genome-wide association, iPSC-RPE

## Abstract

We evaluate whether human induced pluripotent stem cell-derived retinal pigment epithelium (iPSC-RPE) cells can be used to prioritize and functionally characterize causal variants at age-related macular degeneration (AMD) risk loci. We generated iPSC-RPE from six subjects and show that they have morphological and molecular characteristics similar to those of native RPE. We generated RNA-seq, ATAC-seq, and H3K27ac ChIP-seq data and observed high similarity in gene expression and enriched transcription factor motif profiles between iPSC-RPE and human fetal RPE. We performed fine mapping of AMD risk loci by integrating molecular data from the iPSC-RPE, adult retina, and adult RPE, which identified rs943080 as the probable causal variant at *VEGFA*. We show that rs943080 is associated with altered chromatin accessibility of a distal ATAC-seq peak, decreased overall gene expression of *VEGFA*, and allele-specific expression of a non-coding transcript. Our study thus provides a potential mechanism underlying the association of the *VEGFA* locus with AMD.

## Highlights

• iPSC-RPE have morphological and molecular profiles similar to human fetal RPE
• RPE molecular data integration can efficiently prioritize variants at AMD risk loci
• rs943080 is the probable causal AMD risk variant in the *VEGFA* locus
• rs943080 is a regulatory variant associated with lower gene expression of *VEGFA*

## Graphical Abstract

**Figure:**
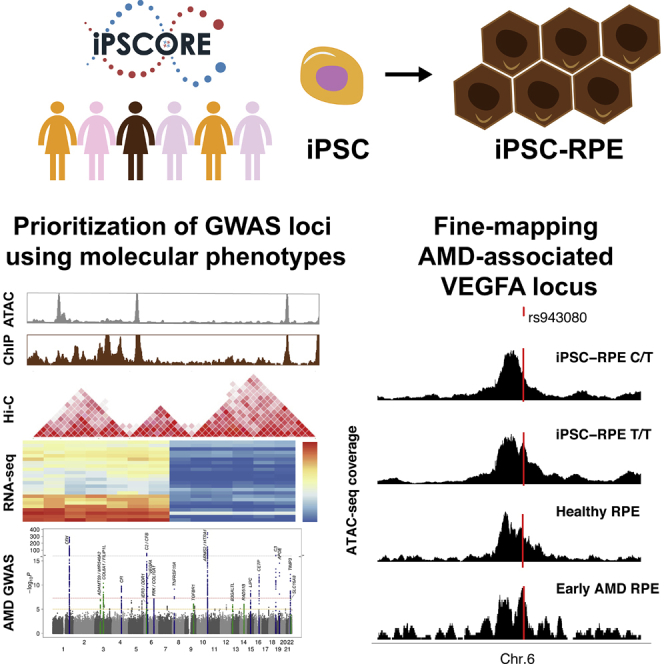

## Teaser

Smith, D'Antonio-Chronowska, and colleagues integrate newly generated epigenetic and transcriptomic data from iPSC-derived retinal pigment epithelium with data from adult samples to prioritize potential causal variants associated with age-related macular degeneration. They prioritize rs943080 at the *VEGFA* locus and show that the risk allele may reduce *VEGFA* gene expression by altering activity of a distal regulatory region.

## Introduction

Age-related macular degeneration (AMD) is a leading cause of vision loss that affects 1.6 million people over the age of 50 years in the United States (CDC, 2018) and has limited therapeutic options (Al-Zamil and Yassin, 2017). Disease development manifests in progressive degeneration in response to oxidative stress and inflammation of the retinal pigment epithelium (RPE) (Kinnunen et al., 2012), a monolayer consisting of a few million cells most densely located in the macula of the eye (Panda-Jonas et al., 1996). AMD has a strong genetic component (Seddon et al., 2005), and through a large international study of 16,144 AMD cases and 17,832 controls, 52 independent AMD risk variants mapping to 34 AMD-associated loci have been identified (Fritsche et al., 2016). As in many common diseases (Gallagher and Chen-Plotkin, 2018, Gusev et al., 2014, Maurano et al., 2012, Visscher et al., 2017), the majority of these loci have strongly associated variants in non-coding regions of the genome, suggesting that they may act through gene regulation. Regulatory variants that affect human disease, such as the variant that affects *IRX3* expression in obesity (Smemo et al., 2014), can have strong effects, but it can be challenging to identify causal distal regulatory variants and link them with their target genes. Indeed, for AMD, while some candidate target genes have been identified (Fritsche et al., 2016), the causal variants and the downstream processes by which they mediate their effects are generally unknown.

Regulatory genetic variation is often cell-type specific and can be studied through genetic analysis of molecular traits such as gene regulation and expression (Albert and Kruglyak, 2015). However, characterizing genetic variation in human RPE is challenging because the number of RPE cells in the human eye is limited (Panda-Jonas et al., 1996), can be affected by lifetime environmental exposures, and requires invasive procedures to collect samples. Induced pluripotent stem cell-derived RPE (iPSC-RPE) is a promising alternative to human RPE for genetic studies as a virtually unlimited number of cells can be obtained with the same genetic background non-invasively. iPSC-RPE has been shown to display characteristics of mature human RPE including polygonal and pigmented morphology, polarity of protein expression and secretion, phagocytosis of photoreceptor outer segments, and maintenance of RPE phenotypes after transplantation into mouse retina (Maruotti et al., 2015). Additionally, stem cell-derived RPE have been effectively transplanted into rodent and primate models, supporting their relevance *in vivo* (Davis et al., 2017, Kamao et al., 2014, Stanzel et al., 2014). Thus, iPSC-RPE could be an effective model system for functionally characterizing regulatory variation associated with AMD.

The identification and functional characterization of causal genetic variants has been improved through fine-mapping algorithms that can incorporate diverse epigenetic annotations. For example, accessible chromatin and active regulatory regions such as promoters and enhancers marked by histone 3 lysine-27 acetylation (H3K27ac) have been shown to be enriched for genetic variants associated with human diseases in cell types relevant for disease and can improve the prioritization of genome-wide association study (GWAS) causal variants through fine-mapping strategies (Pickrell, 2014). Additionally, while many GWAS loci harbor genes that have been implicated in AMD, such as *VEGFA*, which encodes the vascular endothelial growth factor (VEGF) protein that is targeted by three current treatments for AMD (Gragoudas et al., 2004, Heier et al., 2012, Rosenfeld et al., 2006), the causal risk variant and the mechanism of increased disease risk is not known. Thus, the molecular characterization of gene expression and regulatory regions in iPSC-RPE could improve fine mapping of AMD and lead to insights into mechanisms underlying genetic risk variants.

To investigate the utility of iPSC-RPE as a model system to characterize AMD risk variants, we generated iPSC-RPE from six human subjects and integrated gene expression, chromatin accessibility, and H3K27ac chromatin immunoprecipitation sequencing (ChIP-seq) data with complementary published data from human adult subjects to identify potential causal variants at AMD risk loci. We show that the iPSC-RPE shows morphological and molecular characteristics that are similar to those of native RPE including a characteristic polygonal shape, strong melanin pigmentation and expression, and strong zonula occludens 1 (ZO-1), bestrofin 1 (BEST1), and microphthalmia-associated transcription factor (MITF) immunostaining. We show that iPSC-RPE gene expression profiles are highly similar to that of human fetal RPE, and that their ATAC-seq (assay for transposase-accessible chromatin using sequencing) peaks are enriched for relevant transcription factor motifs. We performed fine mapping of AMD risk loci integrating the molecular data from iPSC-RPE, human fetal RPE, and published human retina and RPE samples. At one locus, *VEGFA*, we show that the rs943080 risk allele is associated with regulatory protein binding in iPSC-RPE in a potentially disease-dependent manner, and that the risk allele results in decreased overall *VEGFA* expression, potentially through regulation by a non-coding transcript. These results establish a molecular hypothesis for the *VEGFA* genetic risk locus on AMD and illustrate the potential of iPSC-RPE as a model system to study the molecular function of genetic variation associated with AMD.

## Results

### Derivation of iPSC-RPE

**Figure 1:**
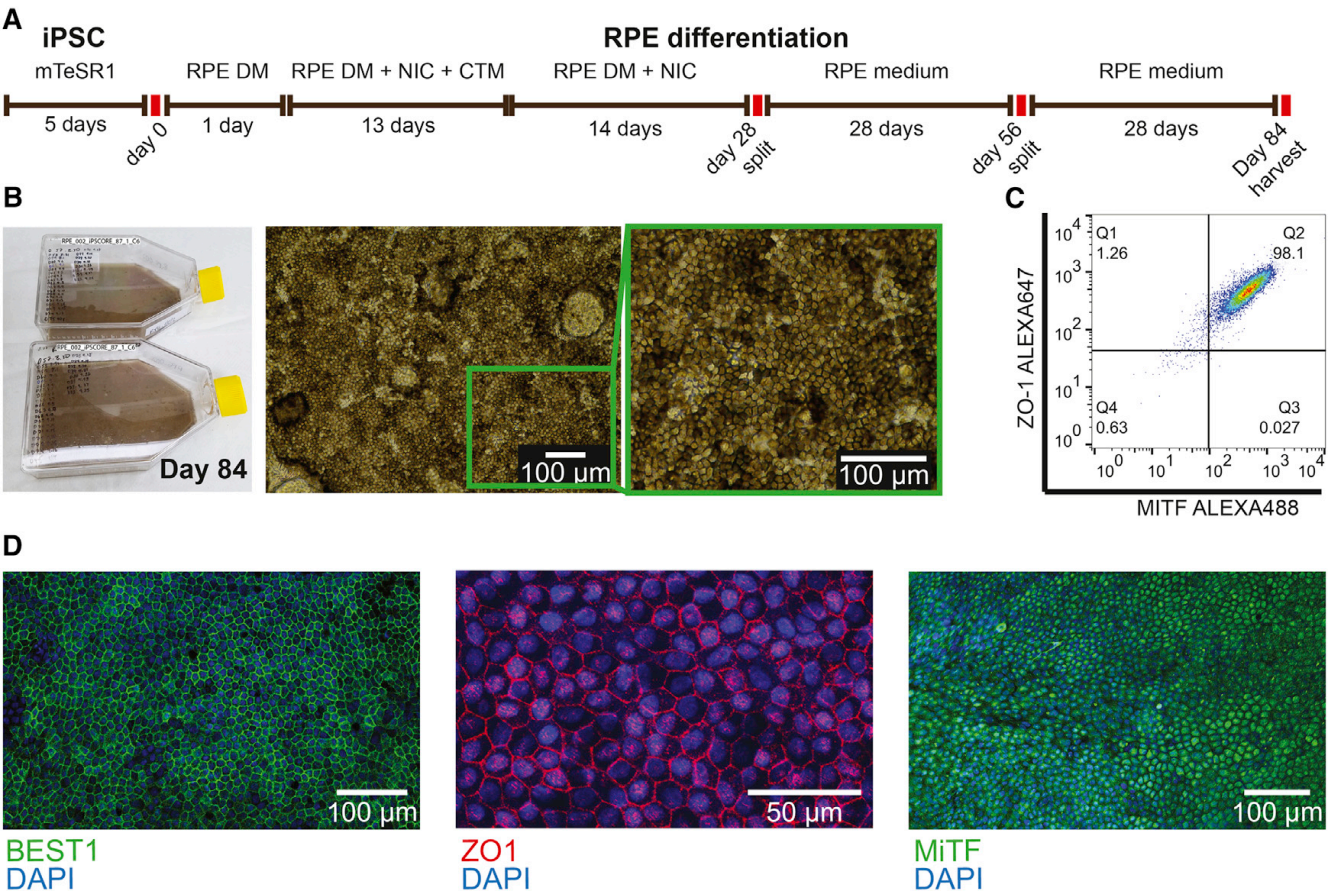
Differentiation of iPSC-RPE (A) iPSC-RPE differentiation protocol. (B) T150 flasks containing iPSC-RPE (iPSCORE 87_1) at day 84 (left). Bright-field image of iPSC-RPE sample (iPSCORE_42_1) at day 84 illustrating a highly organized monolayer with strong melanin pigmentation and characteristic polygonal shape (right). (C) Flow-cytometry analysis of iPSC-RPE (iPSCORE_42_1) at day 84 showing high co-staining of zonula occludens 1 (ZO-1) and microphthalmia-associated transcription factor (MITF). (D) Immunofluorescence analysis of bestrofin 1 (BEST1) (iPSCORE_29_1), ZO-1 (iPSCORE_29_1), and MITF (iPSCORE_42_1).

To derive iPSC-RPE, we chose six induced pluripotent cell (iPSC) lines from unrelated individuals of African American (1), European (3), and East Asian (2) ancestry in iPSCORE that have been established as being pluripotent with low levels of somatic variation (Panopoulos et al., 2017). We then applied a slightly modified version of a protocol validated by Maruotti et al. (2015) (Figure 1A) to differentiate iPSC-RPE. The first pigmented foci of characteristic polygonal cells appeared after ∼2–3 weeks, and virtually all cells were strongly pigmented by day 84 (Figure 1B). We assessed the purity of five out of six iPSC-RPE samples at day 84 by flow cytometry and observed that a high fraction of cells were ZO-1 and/or MITF positive (ZO-1^+^ mean = 93.8% and range = 85.8%–99.4%; MITF^+^ mean = 99.0% and range = 98%–99.8%; ZO-1^+^MITF^+^ mean = 91.5% and range = 87.2%–98.1%) (Figure 1C and Table S1). We also examined two of the iPSC-RPE samples for characteristic RPE marker proteins and observed membrane expression of ZO-1 and BEST1 and nuclear expression of MITF (Figure 1D). Thus, the iPSC-RPE displayed many of the morphological and molecular characteristics of RPE, confirming the robustness of the differentiation protocol.

### The Transcriptomes of Human iPSC-RPE Are Similar to those of Fetal RPE

**Figure 2:**
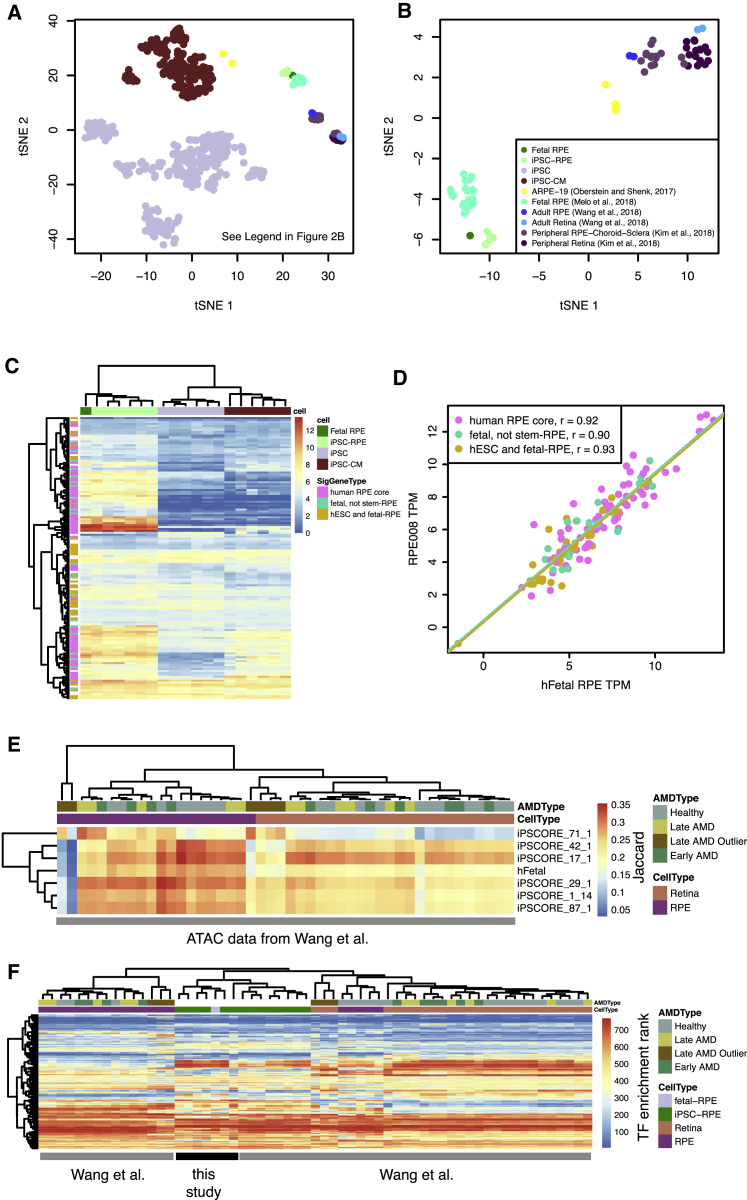
Gene Expression and Chromatin Accessibility of iPSC-RPE and Other Eye Samples (A) tSNE plot based on the first 30 principal components of RNA-seq using 10,000 of the most variable genes. Samples include iPSCs, iPSC-CMs, iPSC-RPE, and fetal RPE data generated for this paper, and RPE and retina samples from external public data sources (see legend in B, inset). (B) tSNE plot based on the first 30 principal components of RNA-seq using 10,000 of the most variable genes. In this case only samples related to RPE and retina are included. (C) Heatmap showing expression levels of RPE signature genes (Strunnikova et al., 2010) in fetal RPE, iPSC-RPE, iPSC, and iPSC-derived cardiomyocytes. Genes are labeled by their gene group as annotated in Liao et al. (2010). (D) Scatterplot showing RNA-seq TPM for an iPSC-RPE (iPSCORE_1_14) compared with the fetal RPE sample for RPE signature genes. Points are color coded according to their classification in Liao et al. (2010) and lines indicate linear regression best fits. (E) Heatmap showing six iPSCORE iPSC-RPE (iPSCORE_42_1, 17_1, 29_1, 1_14, and 87_1) and human fetal RPE ATAC-seq peaks show stronger Jaccard similarity to native RPE ATAC-seq peaks than retina ATAC-seq peaks from adult eyes with and without AMD (Wang et al., 2018). Columns are color coded according to their AMD type and cell type. Three RPE and three Retina samples from the same subject with late AMD were not similar with respect to the other RPE and Retina samples and were labeled as “Late AMD Outlier.” (F) Heatmap showing similarity of transcription factor (TF) binding site enrichment in ATAC-seq peaks across samples. Color indicates the TF's rank of enrichment within each sample with red indicating stronger enrichment. Columns are color coded according to their AMD type and cell type. See also Figure S1.

We first compared iPSC-RPE gene expression with fetal RPE gene expression to examine their similarity overall and at previously established RPE signature genes. We generated RNA sequencing (RNA-seq) data for the six iPSC-RPE, as well as one RPE obtained from a human fetal sample (Table S1). Using principal component analysis (PCA) followed by t-distributed stochastic neighbor embedding (t-SNE), we examined global trends between the iPSC-RPE and the fetal RPE RNA-seq in contrast to 222 iPSC lines (DeBoever et al., 2017) and 144 iPSC-derived cardiomyocytes (iPSC-CMs) (our unpublished data) for which we have previously generated RNA-seq, as well as published RNA-seq data for adult RPE (Wang et al., 2018), adult retina (Kim et al., 2018, Wang et al., 2018), peripheral RPE-choroid-sclera (Kim et al., 2018), fetal RPE (Melo et al., 2018), and the ARPE-19 cell line (Oberstein and Shenk, 2017). When analyzed jointly, we observed that iPSC-RPE grouped with the fetal RPE samples, the adult RPE clustered with the peripheral RPE-choroid-sclera, and the adult retina clustered with the peripheral retina (Figure 2A). These clustering groups were similar when the iPSC and iPSC-CM samples were excluded (Figure 2B). We additionally analyzed gene ontology (GO) enrichments of genes with strong principal component (PC) weights and observed enrichment for a number of RPE functions including sensory organ development (GO:0007423, p = 5.2 × 10^−11^, PC3) and sensory perception of light stimulus (GO:0050953, p = 2.0 × 10^−19^, PC4) (Figure S1 and Table S2). We also found that genes associated with PC3 and PC4 were significantly enriched for previously identified RPE signature genes (Strunnikova et al., 2010) (p = 4.6 × 10^−7^ and 3.8 × 10^−20^, respectively) and the expression patterns of these signature genes in iPSC-RPE were similar to that of the fetal RPE (Figure 2C). As a subset of these signature genes has been previously reported to be different between fetal RPE and stem cell-derived RPE (Liao et al., 2010), we examined their clustering pattern, but did not observe differential clustering (Figure 2C, “fetal, not stem-RPE”). It is possible that the high genomic integrity of the currently studied iPSC lines or improvements to the differentiation protocol (foci formation versus a continuous monolayer) could have resulted in iPSC-RPE that were more similar to fetal RPE. Furthermore, gene expression was highly correlated between fetal RPE and iPSC-RPE across RPE signature genes (average r = 0.90, cor.test p < 1 × 10^−6^, example shown in Figure 2D), suggesting that iPSC-RPE have transcriptomes that are highly similar to those of fetal RPE.

### Chromatin Accessibility of iPSC-RPE

We next examined whether the chromatin accessibility profiles of the iPSC-RPE were similar to those of fetal or adult RPE. We generated ATAC-seq data for the six iPSCORE iPSC-RPEs, and for the fetal RPE (Table S1), and compared them with published ATAC-seq data for RPE and whole retina tissues from adults with and without AMD (Wang et al., 2018). We compared the overlap of the accessible chromatin regions in iPSC-RPE or fetal RPE with adult RPE from both control and AMD subjects using the Jaccard similarity metric and observed high similarity (Figure 2E). We noted, however, that samples from the Wang et al. (2018) study derived from subject “Donor 1” appeared to be outliers with respect to the rest of the data. We therefore labeled these six samples as “Late AMD Outlier.” We also observed that one sample in our study (iPSCORE_71_1) had a lower number of called peaks than the other iPSC-RPE (Table S1) and showed lower similarity to the data from Wang et al. (2018), indicating that it had overall lower quality. However, as this sample was still more similar to the RPE samples than the retina samples, we retained it for downstream analyses. We then examined whether the accessible regions displayed similar transcription factor (TF) motif enrichments across all samples using HOMER (Heinz et al., 2010). We observed high enrichment for TF motifs important for RPE development and function in all RPE samples including OTX2, CRX, and SOX9 (Table S3). We compared the overall pattern of TF enrichment of the fetal and iPSC-RPE with those from Wang et al. (2018) by clustering the profiles by their enrichment rank in order to account for variation in binding specificity across studies. We observed that the iPSC-RPE from this study were most similar to the human fetal RPE and iPSC-RPE samples in Wang et al. (2018) (Figure 2F). However, we observed that the fetal and stem cell-derived RPE showed a number of differences from both the adult RPE and retina samples. These results suggest that iPSC-RPE show a fetal-like regulatory program that is similar to, but distinct from adult RPE and adult retina.

### Enrichment of Regulatory Regions for AMD Genetic Risk

**Figure 3:**
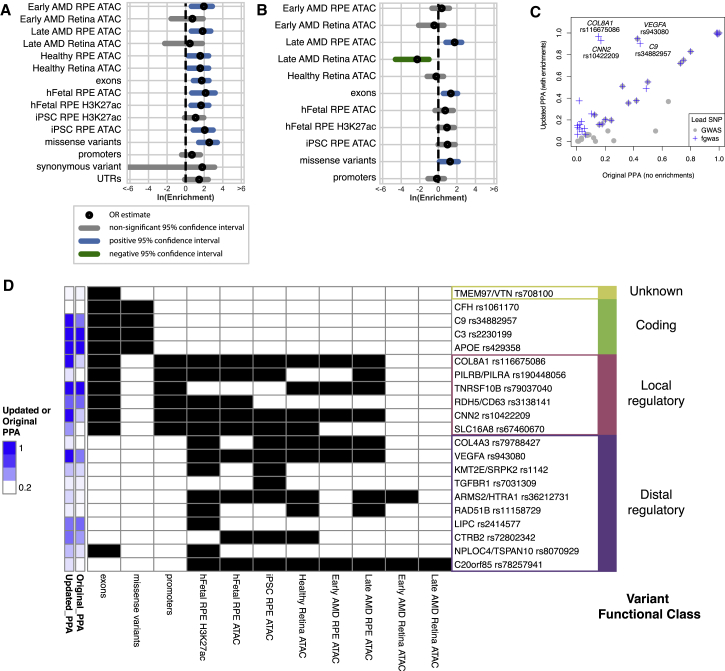
Fine Mapping of AMD GWAS Loci (A) fgwas single enrichment of GWAS signal for multiple genome annotations. Black circles indicate ln(OR) and blue or gray lines show 95% CIs. Blue indicates a significant positive enrichment. (B) Final combined model used by fgwas to prioritize variants. Black circles indicate ln(OR) and blue, gray, or green lines show 95% confidence intervals (colored to indicate significance different from 0; green negative, blue positive). Note that in this combined model, associations reflect independent associations for each annotation and exclude annotations that did not significantly improve the model, and so differ from single enrichments shown in (A). (C) Scatterplot showing posterior probability of association (updated PPA) versus original posterior probability of association (original PPA) for GWAS loci. GWAS lead SNPs are shown in gray. SNPs that were chosen as variant at the locus with the highest PPA by fgwas are shown in blue. When the variant with the highest updated PPA is the GWAS lead variant, the points overlap. The four variants where the fgwas prediction improved the PPA from <0.5 to >0.8 are labeled by their candidate gene and fgwas lead SNP. (D) Heatmap indicating the presence (black) or absence (white) of each annotation for the 21 lead fgwas variants that were prioritized by the model. The original and updated PPAs are shown in blue and are the same as in (C). Variants are grouped into variant functional classes according to whether they were unknown, coding, local regulatory (located in promoters), or contained in distal regulatory (ATAC-seq or H3K27ac without promoter) annotations. See also Figure S2.

To establish whether regulatory regions in iPSC-RPE could be used to help identify potential causal variants for AMD, we generated H3K27ac ChIP-seq data from the iPSC-RPE and tested these and the chromatin accessibility regions for enrichment of AMD genetic risk (Fritsche et al., 2016) using fgwas (Pickrell, 2014), a statistical framework that integrates functional genomics annotations and GWAS summary statistics to identify putative causal variants at known loci. We found that H3K27ac peak regions from fetal RPE, as well as ATAC-seq peak regions from iPSC-RPE, fetal RPE, adult AMD RPE, adult healthy RPE, and adult healthy retina, were enriched for AMD genetic risk (Figure 3A). In addition, exon regions and missense variants were also enriched. However, ATAC-seq regions from adult AMD (early and late) retina samples, H3K27ac peak regions from iPSC-RPE, and promoters showed positive associations, but were not significantly enriched. We also observed that the regions from the Late AMD Outlier samples showed depletion for AMD risk and we therefore excluded these samples from downstream analyses (Figure S2). Furthermore, iPSC-RPE ATAC-seq and H3K27ac ChIP-seq regions from subjects of different ethnicities showed overall similar levels of enrichment and were therefore merged (Figure S2). Using the annotations shown in Figure 3A, we created a combined model that removes redundant annotations and assesses the independent risk of each annotation (Figure 3B). We observed that while not all enrichments were significant, fetal, iPSC, and adult RPE samples all provided independent positive information for AMD risk. ATAC-seq regions from retina samples, however, showed negative enrichments, suggesting that the positive associations in the single model were captured by the other annotations and were RPE specific. These results indicate that chromatin accessibility regions in RPE from adults, fetal, and iPSC-derived samples capture complementary risk regions associated with AMD risk and could be used to improve identification of potential causal variants.

### Fine Mapping of AMD-Associated Loci with fgwas

To identify potentially causal variants associated with AMD, we used fgwas to perform fine mapping of 32 of the 34 AMD risk loci for which we were able to obtain sufficient data (see Experimental Procedures) based on the estimates from the combined model in Figure 3B. For each of the 32 risk loci, we calculated 95% credible sets pre- and post-prioritization (Table S4), and found that the prioritization improved the number of variants in the credible set on average from 28.9 to 23.5 (19% reduction). For 9 of the 32 risk loci, we identified a single variant with a posterior probability of association (PPA) >0.8 (Table S4). Of these, four showed strong shifts between their PPAs before and after updating with the enrichments, including three loci where the GWAS lead variant was not the prioritized variant (*COL8A1*, *CNN2*, and *C9*), and one where the lead variant was the prioritized variant (*VEGFA*) (Figure 3C).

To obtain insight into the types of regulation that could be associated with each of the 32 GWAS loci, we examined how the top variants prioritized by fgwas were associated with each annotation and classified each into putative functional classes according to their annotation patterns (Figure 3D). Of the 32 fgwas lead variants, 21 were associated with an annotation. The majority of the variants (16/21 [76%]) were located in regulatory regions, overlapping both promoter (local regulatory, 6/21 [29%]) and proximal regions (distal regulatory, 10/21 [48%]). An additional four were missense variants (coding, 4/21 [19%]). One variant was located in an exon (in a 3′ UTR), but was not associated with other annotations, and was classified as unknown (1/21 [5%]). We observed 13 regulatory variants with ATAC-seq annotations present in either iPSC-RPE or adult AMD RPE (early- or late-stage AMD), of which seven were present in both sample types. The remainder were present only in iPSC-RPE (4/13) or only in adult AMD RPE (2/13), suggesting that the majority of ATAC-seq regions associated with AMD GWAS loci are present in iPSC-RPE, but that in some cases they are only present in adult RPE. Overall, these results establish a set of prioritized variants at AMD loci, identify four variants with high PPA that were not previously distinguished from other variants at their loci, and illustrate that iPSC-RPE and adult RPE provide overlapping but complementary regulatory annotations.

### rs943080 Is Associated with Regulatory Effects on *VEGFA* Expression

**Figure 4:**
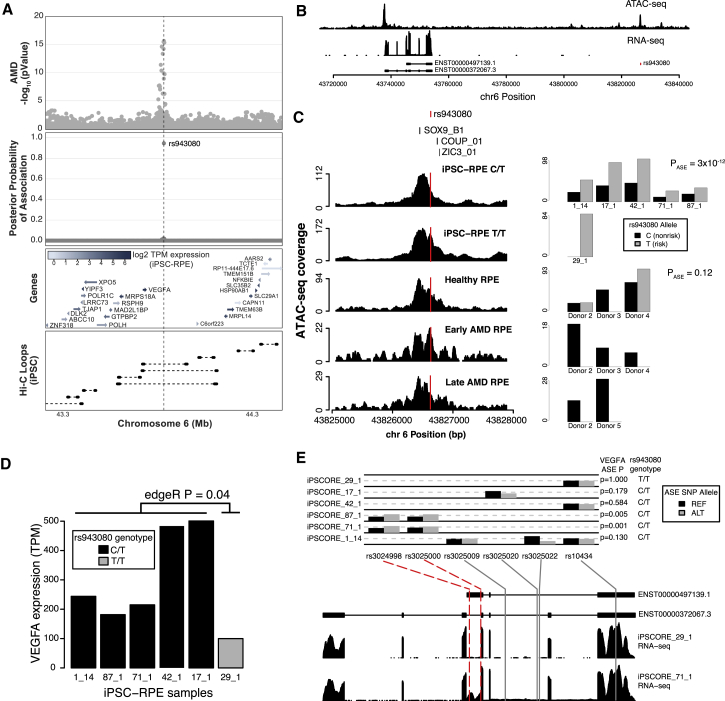
The rs943080 SNP Is Associated with Expression of *VEGFA* and Allele-Specific Expression of a Non-coding *VEGFA* Transcript (A) Genomic region surrounding the genome-wide significant lead SNP near *VEGFA* showing AMD GWAS association (top), posterior probability of causal association from fgwas for rs943080 (second from top), gene location and expression level in iPSC-RPE (third from top), and Hi-C chromatin loops measured in iPSCs (bottom). (B) Wig-coverage plots showing example ATAC-seq and RNA-seq data from iPSC-RPE. Two *VEGFA* transcripts are shown and the location of rs943080 is indicated. (C) rs943080 shows allele-specific expression of an ATAC-seq peak. TF motifs are shown at the top of example wig coverage plots from each type of RPE samples (iPSC-RPE that is heterozygous for rs943080, iPSC-RPE that is homozygous risk for rs943080, RPE from normal adult eyes, RPE from AMD eyes with early-stage disease, and RPE from AMD eyes with late-stage disease); the red line indicates the location of rs943080 in the peaks (left). For each sample, the number of reads for the reference (non-risk) and alternative (risk) alleles are shown as a bar plot; for heterozygous samples, the p value corresponds to a meta-analysis of tests of ASE using a binomial test (right). For data from Wang et al., subjects are named Donors 1–5 within the Healthy group and Donors 1–5 within the AMD group. Samples from subjects with AMD can fall into either Early or Late AMD, depending on pathology. (D) Bar plot showing overall TPM gene expression for *VEGFA* in each of the iPSC-RPE samples. Bars are color coded by sample genotype, and the edgeR p value for the difference between genotype groups is shown. (E) ASE of *VEGFA* in the iPSC-RPE. Bar plots showing the allele frequencies for six SNPs covering the *VEGFA* gene in each sample where they are heterozygous; ASE p values for overall gene expression from MBASED are listed for each sample on the right (top). The location of each of the SNPs relative to *VEGFA* transcripts and wig coverage plots of RNA-seq data from a homozygote risk (iPSCORE_29_1) and heterozygote (iPSCORE_71_1) individual are shown (bottom); red dotted lines highlight the two SNPs that overlap an exon of a non-coding transcript (ENST00000497139.1) and are driving the significant ASE association in two individuals.

To examine the potential function of a SNP with a high posterior probability of causality, we further examined the molecular data associated with rs943080 at the *VEGFA* locus. *VEGFA* is a strong candidate for the regulatory target of this SNP as it is highly expressed in RPE and the SNP is within a Hi-C chromatin loop identified in iPSCs (Greenwald et al., 2019) associated with the *VEFGA* promoter (Figure 4A). The rs943080 SNP is located ∼90 kb from the start position of the *VEGFA* gene in a strong iPSC-RPE ATAC-seq peak (Figure 4B). While the SNP does not interrupt a TF motif or appear to create a new motif (data not shown), the peak overlaps three motifs of TFs relevant for the eye and/or RPE (SOX9 [Tang et al., 2015], COUP-TFI/II [Tang et al., 2015], and ZIC3 [Zhang et al., 2004]; Figure 4C); additionally all three TFs were expressed in iPSC-RPE (data not shown). In addition, COUP-TFII has been implicated in angiogenesis and regulation of VEGF receptor in the cardiovascular system (Pereira et al., 1999), suggesting that it could also play a role in angiogenesis in RPE. We first examined whether the ATAC-seq peak showed allele-specific expression at the rs943080 SNP. For the six subjects for which we obtained iPSC-RPE, we used whole-genome sequence data to determine that five were heterozygous for the rs943080 SNP (C/T) and one was homozygous for the risk allele (T/T). Across the five heterozygous subjects, we observed a significant allele-specific effect (ASE) for the risk allele (p = 3 × 10^−12^, Figure 4C), indicating that the risk allele was associated with stronger chromatin accessibility. These results suggest that rs943080 may act through allele-specific regulation of the distal ATAC-seq peak.

To examine whether adult RPE samples also showed ASE, we inspected allelic depth in the samples from Wang et al. (2018). We observed reads for both alleles for two of the three adult healthy RPE subjects, consistent with these individuals being heterozygous for the variant, but did not observe significant ASE in these samples (p = 0.12), potentially due to low comparative read depth. In the three adult early-stage and two adult late-stage AMD RPE samples from four subjects, we failed to observe any reads with the risk allele, which could be explained because (1) they are all homozygous for the non-risk genotype or (2) in AMD samples there is strong ASE for the non-risk allele. While we do not have genotype data for these four subjects, given the risk allele frequency (0.52 in European populations, higher in other populations and in AMD subjects), it is unlikely that they would all be non-risk homozygotes (probability = 0.003). Therefore, it is possible that the rs943080 variant could show ASE of chromatin accessibility favoring the non-risk allele in RPE from AMD subjects, in contrast to the ASE of chromatin accessibility favoring the risk allele in iPSC-RPE and adult healthy RPE. These results therefore suggest that rs943080 is associated with differential chromatin accessibility in iPSC-RPE and healthy adult RPE, but that it may act differently in an AMD context.

To examine whether the rs943080 SNP was also associated with *VEGFA* gene expression, we examined overall expression levels and ASE in RNA from the iPSC-RPE samples. We observed that the five heterozygous samples showed significantly higher overall gene expression than the one homozygous risk sample (edgeR p = 0.04, Figure 4D), suggesting that the rs943080 risk allele may drive decreased *VEGFA* gene expression in RPE. Of note, rs94308 is not an expression quantitative trait locus for any GTEx tissues (GTEx Consortium et al., 2017), but this could be due to the fact that the interval around the SNP is only annotated as an enhancer region in Roadmap (Roadmap Epigenomics et al., 2015) in a small number of stem cell or fetal tissues. Moreover, the five heterozygous samples showed expression heterogeneity within them, suggesting that additional factors also contribute to variation in *VEGFA* expression.

We then further examined the transcriptome data and identified two samples with significant ASE for *VEGFA* RNA (iPSCORE_87_1 and iPSCORE_71_1, Figure 4E). To determine whether the ASE was associated with a specific region of the *VEGFA* transcripts, we examined the locations and frequencies of the SNPs that were used to estimate ASE. We found that the ASE for these two subjects was driven by two SNPs that were located in an exon of a non-coding transcript (ENST00000497139.1) and which did not overlap coding exons, suggesting that the ASE observation could be restricted to the non-coding transcript. Consistent with this model, we observed that a third SNP located in the 3′ UTR, which is shared across almost all *VEGFA* transcripts, had high coverage in multiple samples and was not associated with ASE. Thus, while rs943080 is associated with an overall decrease in *VEGFA* expression, it may be mediated not through ASE of all transcripts but rather through a specific non-coding transcript.

## Discussion

In this study, we have generated RNA-seq, ATAC-seq, and H3K27ac ChIP-seq data from six human iPSC-RPE differentiations as well as data from one human fetal RPE sample. We integrated this data with published ATAC-seq data from RPE and retina from adults with and without AMD to prioritize specific variants at AMD risk loci. The majority of the variants whose prioritizations were altered by our model were located in regulatory regions, although some were also coding variants in genes. Overall, we observed strong improvements in the prioritization of four variants, including a potential regulatory SNP (rs943080) near *VEGFA* and a coding SNP (rs34882957, pP167S) near the *C9* gene. While rs34882957 would not be expected to alter gene expression or regulation, it has recently been shown to be associated with increased serum concentration (Geerlings et al., 2017) and *in vitro* with spontaneous polymerization (Kremlitzka et al., 2018), providing additional support that the variant has functional effects on C9 that could be involved in mediating AMD risk.

By examining RNA-seq and ATAC-seq data from individuals with varying genotypes at the rs943080 SNP near *VEGFA*, we suggest that the risk variant may result in decreased gene expression of a non-coding transcript of *VEGFA* in developing or normal RPE cells. While this transcript has not been well characterized, it is highly expressed in many tissues (Gamazon et al., 2018) and could have a regulatory role as it includes the 3′ UTR, which has been shown to have regulatory effects on translation (Ray et al., 2009). Of note, there was one additional variant in the *VEGFA* 95% credible set (rs7758685, PPA = 0.02) that could also be considered a candidate for being causal, as it shared the same genotypes as rs943080 in the six samples and so was equivalently associated with *VEGFA* gene expression. rs7758685 did not, however, occur in a regulatory region and we therefore could not perform an ATAC ASE analysis to access its functionality. Given the strong LD with rs7758685 and the fact that the strength of significance with altered gene expression was modest, future experimental studies in appropriate model systems are required to definitively show that the rs943080 SNP causally affects protein binding in RPE and gene expression of *VEGFA*.

Our findings are relevant for the treatment of AMD, as anti-VEGF is the only currently approved treatment for “wet” AMD that shows choroidal neovascularization. While this treatment improves symptoms that are driven by the growth of blood vessels, such as swelling and bleeding, it is not clear that it stops progression of the disease, and it may be associated with the further development of geographic atrophy seen in “dry” AMD (Enslow et al., 2016). Specific knockout of *VEGFA* in RPE in mice has been shown to result in dysfunction and loss of the choriocapillaris and cone photoreceptors, suggesting that low levels of *VEGFA* gene expression could affect AMD pathology (Kurihara et al., 2012). Our results are consistent with this model and suggest that the rs943080 risk variant could act through a reduction of *VEGFA* expression prior to AMD onset. We were also able to examine the chromatin accessibility of this region in previously published ATAC-seq samples from adult RPE with and without AMD. While we did not have access to genetic data for these subjects, we observed biallelic accessibility in healthy RPE samples and monoallelic accessibility in AMD RPE samples with only the non-risk allele expressed. As increased chromatin accessibility in healthy RPE was associated with decreased *VEGFA* expression, it is plausible that a lack of chromatin accessibility in AMD RPE samples could be associated with increased *VEGFA* expression. Further study of the transcriptional effects of these variants in subjects with genotype data is therefore needed.

We were able to gain insight into the *VEGFA* locus with relatively few iPSC-RPE samples because we had a number of heterozygous individuals as well as an individual who was homozygous for the rs943080 risk allele. However, for GWAS loci with many candidate target genes, larger numbers of iPSC-RPEs will be required to characterize ATAC ASE and changes in gene expression more generally. Indeed, subsequent analyses of variants in the 95% credible sets for the *COL8A1* and *CNN2* GWAS loci showed that the number of heterozygous sites in the iPSC-RPEs in our study were too few to observe significant ATAC ASE effects. Thus, to gain insight into functional genetic variation genome wide, it will likely be necessary to obtain data for hundreds of samples. As high-quality RPE samples are challenging to obtain from human cadavers and because sample limitations may restrict molecular characterization, iPSC-RPE could potentially be generated from the hundreds of individuals needed. These data suggest that iPSC-RPE are well suited for the genetic characterization of functional variation in RPE and that these large studies could aid in the identification of causal variants at AMD risk loci.

## Experimental Procedures

Please refer to Supplemental Experimental Procedures for detailed methods.

### Sample Information

We obtained iPSC lines from iPSCORE (Panopoulos et al., 2017) (Table S1) that were of diverse ancestries (3 European, 2 East Asian, and 1 African American), age of donation, and were female. The recruitment of these individuals was approved by the Institutional Review Boards of the University of California, San Diego, and The Salk Institute (project no. 110776ZF). Human fetal RPE cells were purchased from Lonza.

### RPE Derivation and Characterization

To obtain iPSC-RPEs, we cultured iPSCs as a monolayer on Matrigel in mTeSR1 medium. Once cells reached the desired confluence, mTeSR1 medium was replaced with RPE differentiation medium (RPE DM) and cells were cultured for 24 h. After 1 day, RPE DM was supplemented with 10 mM nicotinamide (NIC) and 50 nM chetomin. After 2 weeks, cells were cultured in RPE DM medium supplemented with 10 mM NIC and then split at day 28 and day 56 with culturing in RPE medium until day 84.

### Cellular Data Generation

#### Flow-Cytometry Analysis

RPE were analyzed for ZO-1 and MITF co-expression using flow cytometry. The fractions of ZO-1- and MITF-positive cells were similar across all tested iPSC-RPE lines.

#### Immunofluorescence Characterization

Fresh or cryopreserved iPSC-RPE cells from RPE001 and RPE005 at day 84 were characterized by immunofluorescence using anti-ZO-1 antibody and a mouse monoclonal anti-MiTF antibody or anti-ZO-1 and mouse monoclonal anti-BEST1 antibody.

### Molecular Data Generation and Processing

#### RNA-Seq

mRNA libraries were prepared and sequenced to 40 million 150-bp paired-end reads per sample. RNA-seq reads were aligned using STAR with a splice junction database. Transcript and gene-based expression values were quantified using the RSEM package and normalized to transcript per million base pairs (TPM).

#### ATAC-Seq

We performed ATAC-seq, and libraries were sequenced to 80 million 150-bp paired-end reads. ATAC-seq reads were aligned using STAR to hg19, quality and insert size filtered, and peak calling was performed using MACS2 on BAM files.

#### ChIP-Seq

For H3K27ac, formaldehyde crosslinked iPSC-RPE cells were lysed and sonicated. For each sample, H3K27ac antibody was used for overnight ChIP in IP buffer. Beads with immunoprecipitated chromatin were washed, eluted, and reverse crosslinked. Reverse crosslinking samples were then purified, eluted, and quantified. Libraries were generated, barcoded, and sequenced to 40 million 150-bp paired-end reads. ChIP-seq reads were mapped to the hg19 reference using BWA. Reads were quality filtered and peak calling was performed for each sample using MACS2 (Zhang et al., 2008) with reads derived from sonicated chromatin not subjected to IP used as a negative control.

### Data Analysis

#### RNA-Seq Analysis

PCA was performed on the 10,000 genes with the most variable expression across all analyzed samples. Functional enrichment of genes associated with each PC was performed using goseq. Rtsne was used to perform t-SNE on the first 30 components from the PCA. For each iPSC-RPE, we tested the correlation between the expression of the log_2_(TPM) values for the iPSC-RPE and the fetal RPE sample for each set of RPE signature genes.

#### ATAC-Seq Analysis

ATAC-seq data were obtained from the Gene Expression Omnibus (GEO: GSE99287). The five healthy donors were treated as unique subjects. For the AMD samples, as samples from the same subject could be annotated as early or late depending on the affected status of the eye, the five AMD donors were treated as unique subjects, whether or not the sample was annotated as early or late AMD.

#### TF Enrichment

Enrichment for TF motifs was performed with HOMER using HOCOMOCOv11 motifs. Sequences flanking the summits identified by MACS2 were examined using findMotifsGenome. To cluster TF motif profiles, we ranked the enrichment p values within each sample across all TFs clustered and clustered them using hierarchical clustering.

#### Measuring AMD GWAS Enrichment within Tissue ATAC-Seq Peaks

To measure the enrichment of AMD GWAS within different tissues, we applied fgwas on each set of ATAC-seq peaks independently. Annotations for exonic, promoter, and UTR regions were obtained from GENCODE. Annotations for missense and synonymous variants were obtained from the 1,000 Genomes Project Phase 3. Variants within each set of ATAC-seq peaks or other annotations were input to fgwas, *Z* scores were calculated using the β and its standard error, and fgwas was run using these *Z* scores and standard errors on consecutive ∼1-Mb intervals across the genome.

#### Fine Mapping of AMD GWAS Loci

To perform fine mapping of the AMD GWAS loci, we trained an fgwas model containing annotations from ATAC-seq data, H3K27ac data, and genome annotations. We applied fgwas on these annotations, and used a cross-validation penalty of 0.30. Finally, we removed annotations from the model until the likelihood stopped increasing, resulting in 11 annotations from the three types being retained. We used the model with fgwas to update the Bayes factors for each variant using the cross-validation estimated ridge parameter and calculated the posterior probability of causality for each variant within 1-Mb windows flanking the reported lead variant. The PPA is the proportion of the total GWAS risk signal at a locus measured by Bayes factors that is attributed to a particular variant, multiplied by the probability that the genomic region contained a real signal.

#### *VEGFA* Locus Annotation

To visualize the *VEGFA* region, we plotted −log_10_ p values along with the PPA of all SNPs after prioritization with fgwas. TPM normalized RNA-seq expression data from an iPSC-RPE was used to identify expressed genes in the region.

#### ATAC-Seq Peak ASE of rs943080

To examine ASE at the ATAC-seq peak containing rs943080, we measured allelic read depth. For each subject, an ASE p value was calculated by testing the read depth counts to the expected frequency (50%) using a binomial test. Across all heterozygotes, a meta-analysis p was calculated. For the adult early- and late-stage AMD samples, we calculated the probability of observing four subjects with a homozygous reference genotype as the square of the reference allele frequency in the 1,000 Genomes Project EUR population (0.48) to the fourth power.

#### RNA-Seq Analysis at *VEGFA*

Expression differences between the iPSC-RPE from the five subjects heterozygous for rs943080 were compared with the one homozygous subject. Reads counts were merged across all transcripts to examine gene-level differences. ASE analysis of RNA-seq data was performed, the number of read pairs supporting each allele was counted, and heterozygous single-nucleotide variants (SNVs) were retained if the reference or alternative allele had more than eight supporting read pairs, the reference allele frequency was between 2% and 98%, and the SNV was located in unique mappability regions.

## Author Contributions

Conceptualization, E.N.S., A.D.-C., P.B., and K.A.F.; Software, E.N.S., M.D., W.W.Y.G., and H.M.; Formal Analysis, E.N.S., A.D.-C., M.D., and W.W.Y.G.; Investigation, A.D.-C., V.B., L.R.A., and S.B.; Data Curation, E.N.S., M.D., and H.M.; Writing – Original Draft, E.N.S., A.D.-C., M.D., W.W.Y.G., and K.A.F.; Writing – Review & Editing, R.A., S.B., and R.P.; Visualization, E.N.S., M.D., and W.W.Y.G.; Supervision, E.N.S., R.P., A.D.-C., S.B., and K.A.F.; Project Administration, E.N.S., A.D.-C., R.A., and K.A.F.; Funding Acquisition, S.B., R.A., and K.A.F.

## Accession Numbers

ATAC-seq and RNA-seq data can be accessed through dbGaP: phs000924, the Gene Expression Omnibus (GEO: GSE126847), and through the UCSC Genome Browser (iPSCORE_RPE Public Track Hub). The accession numbers for the additional ATAC-seq and RNA-seq data reported in this paper are GEO: GSE99287, GSE99248, GSE92346, and GSE99454.

## Supplemental Information

Document S1. Supplemental Experimental Procedures and Figures S1 and S2

Table S1: List of Samples and Data Identifiers Used in the Study, Related to Figure 1

Table S2: GO-Term Results for Analyses of PCs 3 and 4, Related to Figure S1

Table S3: Full List of −ln p Values for Transcription Factor Motif Enrichment, Related to Figure 2

Table S4: Full List of Variants in 95% Credible Sets Pre and Post Prioritization by fgwas, Related to Figure 3

Document S2. Article plus Supplemental Information
