# Supplementary material for: Human iPSC-Derived Retinal Pigment Epithelium: A Model System for Prioritizing and Functionally Characterizing Causal Variants at AMD Risk Loci
Source: Stem Cell Reports. 2019 May 9;12(6):1342–53. doi: 10.1016/j.stemcr.2019.04.012 (PMC6565613; doi:10.1016/j.stemcr.2019.04.012)
Supplement: Document S1. Supplemental Experimental Procedures and Figures S1 and S2 [file mmc1.pdf]

Stem Cell Reports, Volume *12*

Supplemental Information

Human iPSC-Derived Retinal Pigment Epithelium: A Model System for Prioritizing and Functionally Characterizing Causal Variants at AMD Risk Loci

Erin N. Smith, Agnieszka D'Antonio-Chronowska, William W. Greenwald, Victor Borja, Lana R. Aguiar, Robert Pogue, Hiroko Matsui, Paola Benaglio, Shyamanga Borooah, Matteo D'Antonio, Radha Ayyagari, and Kelly A. Frazer

Supplemental Figures

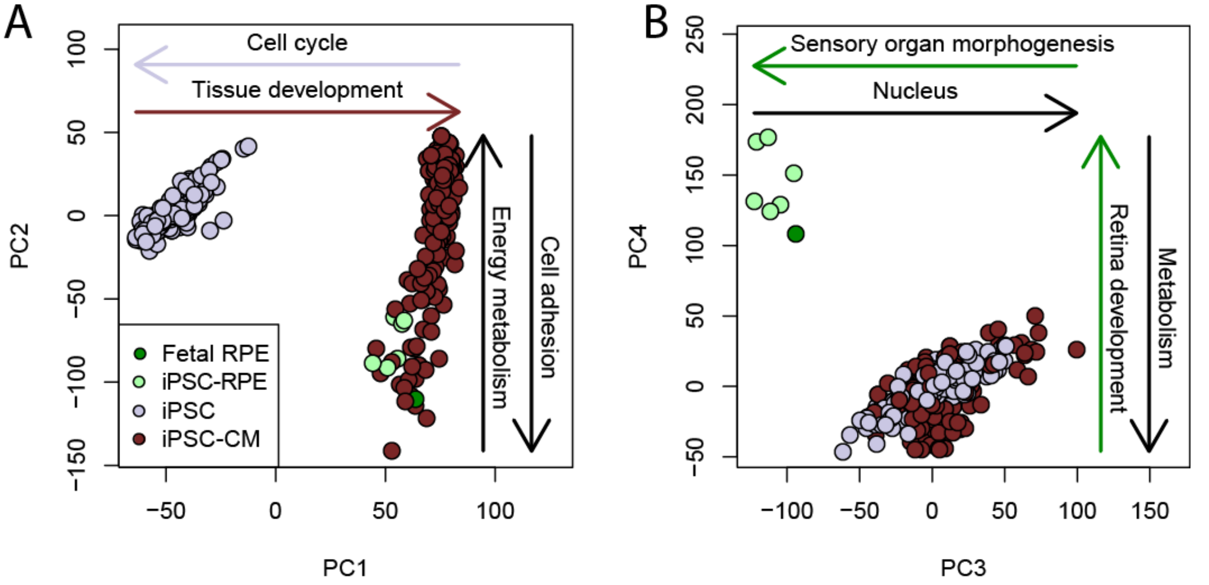

Figure S1: Principal Component Analysis of RNA-seq data, See also Figure 2.

A) PCA plot of first two RNA-seq principal components (PCs) based on RNA-seq from 10,000 of the most variable genes**.** Annotated arrows indicate gene ontology enrichment for genes with in the top or bottom 10% weights for each PC. **B)** PCA of the third and fourth PCs showing the clustering of the iPSC-RPE samples with the fetal RPE sample.

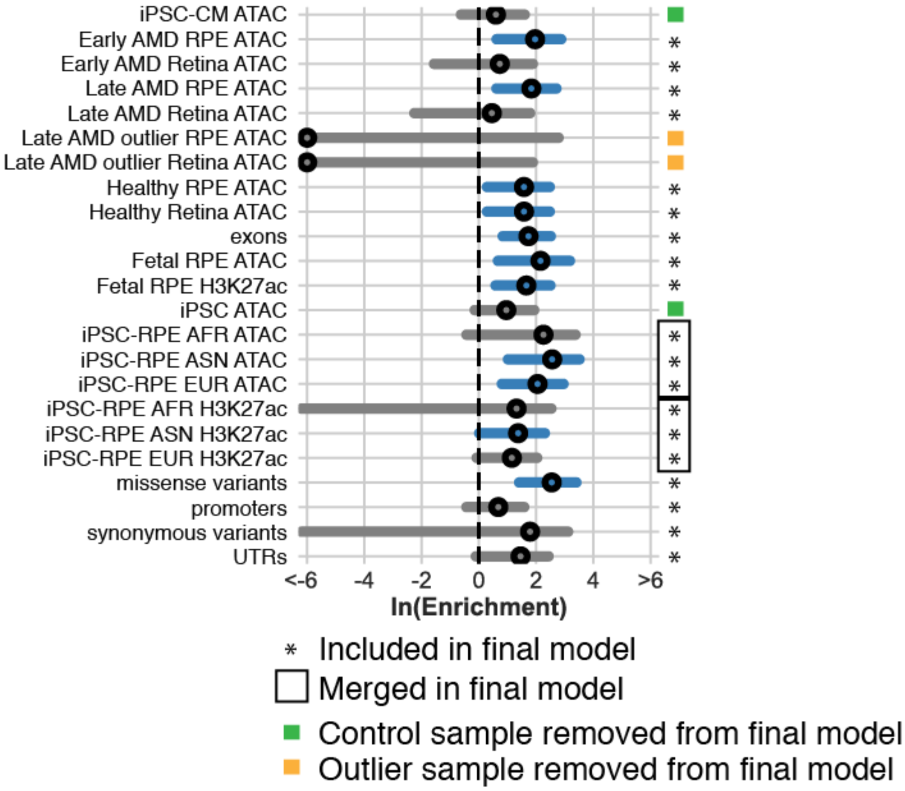

Figure S2. Single fgwas enrichments for annotations including negative controls iPSC ATAC and iPSC-CM ATAC, Late AMD outlier samples, and iPSC-RPE ATAC and H3H27Ac split by ancestry, See also Figure 3.

Asterisks indicate whether the annotation was included in the final model and rectangles around asterisks indicate whether the annotations were merged before being included in the final model. Green rectangles indicate control samples (iPSC and iPSC-CM) that were excluded in the final model. Orange rectangles indicate Late AMD outlier samples (RPE and Retina) that were excluded in the final model as they showed negative enrichments with very low ln(ORs).

Supplemental Experimental Procedures

Sample Information

We obtained iPSC lines from iPSCORE (Panopoulos et al., 2017) (Supplemental Table 1). These were of diverse ancestries (3 European, 2 East Asian, and 1 African American), ranged in age of donation from 21-62 years old, and were female. The subjects were not screened for AMD. Human fetal RPE cells were purchased from Lonza (Cat no: 00195407) and cultured according to manufacturer's recommendations for 56 days until the cells reached 100% confluency and were pigmented after which cells were collected. In detail, cells were washed with PBS, incubated with 1ml of Accutase per well at 37°C for 12 min and from the wells. Cells were filtered using a 70mm strainer and counted. Pellets of cells were collected for the molecular assays and cryopreserved for the future experiments.

RPE derivation and characterization

We obtained iPSC-RPE using a slightly modified version of the Maruotti et al. protocol (Maruotti et al., 2015). The iPSCs were cultured as a monolayer on Matrigel® in mTeSR1 medium. Once cells reached the desired confluency (>80%, approximately 5 days), mTeSR1 medium was replaced with RPE differentiation medium (RPE DM) (24ml/10cm dish) and cells were cultured for 24h. After one day, RPE DM was supplemented with 10mM Nicotinamide (NIC) and 50nM Chetomin (CTM; a strong inducer of RPE (Maruotti et al., 2015)) (RPE DM + NIC + CTM). After 2 weeks, cells were cultured in RPE DM medium supplemented with 10mM NIC (RPE DM + NIC). The cells were then split at day 28 and day 56 with culturing in RPE medium until day 84. About one week after each passage, the polygonal cells formed a very tight, fully pigmented monolayer. Cells were visually examined at day 84 using an EVOS XL Core microscope. Of note, at day 84 we were able to cryopreserve, thaw and then further expand the iPSC-RPE.

Cellular Data Generation

Flow cytometry analysis

RPE were analyzed for ZO-1 and MITF co-expression using flow cytometry. iPSC-RPE cells at day 84 were collected from T150 flasks as described above, filtered using a 70μm strainer and counted. $5x10^6$ cells were fixed and permeabilized using the Fixation/Permeabilization Solution Kit with BD GolgiStop™ (BD Biosciences 554715)

following manufacturer recommendations, and resuspended at the concentration of $1x10^7$/ml. $2.5x10^5$ fixed and permeabilized cells were stained in 1X BD Perm/Wash™ Buffer with a Rabbit polyclonal anti-ZO-1 antibody (Abcam, ab59720; 1:10), Mouse monoclonal anti-MiTF antibody (Abcam, ab12039, 1:10), or appropriate class control antibodies for 1h at room temperature followed by a Donkey-anti-Rabbit AlexaFluor 647 conjugated antibody (Abcam, ab150075; 1:200) or Goat-anti-Mouse AlexaFluor 488 conjugated antibody (Thermo Scientific, A-11001; 1:200). Cells were acquired using FACSCanto II (BD Biosciences) and analyzed using FlowJo software V 10.4. The fraction of ZO-1 and MITF positive cells were similar across all tested iPSC-RPE lines (ZO-1+ mean = 93.8% and range = 85.8-99.4%; MITF+ mean = 99.0% and range = 98-99.8%; ZO-1+MITF+ mean = 91.5% and range = 87.2-98.1), confirming the robustness of the protocol.

Immunofluorescent characterization

Fresh or cryopreserved iPSC-RPE cells from RPE001 and RPE005 at day 84 were thawed and plated on Matrigel® coated glass Millicell EZ SLIDE 8-well glass slides (Millipore, PEZGS0816) and cultured for 10 days in RPE medium. The cells were then fixed with 4% PFA for 10 min at room temperature (RT), washed twice with PBS, 0.1% Tween® 20, incubated for 20min at room temperature in a blocking – permeabilizing solution (PBS, 1% BSA, 0.1% Triton X-100) and stained overnight at 4°C with either a Rabbit polyclonal anti-ZO-1 antibody (Abcam, ab59720; 1:250) and a mouse monoclonal anti-MiTF antibody (Abcam, ab12039, 1:100) or anti-ZO-1 and mouse monoclonal anti-Bestrophin 1 antibody (Novus Biologicals, NB300-164SS; 1:150) or with the appropriate class control antibodies. The next day, cells were washed three times with PBS and subsequently incubated for 1h at RT with a Goat-anti-Mouse AlexaFluor 488 conjugated antibody (Thermo Scientific, A-11001; 1:250) or Donkey-anti-Rabbit AlexaFluor 647 conjugated antibody (Abcam, ab150075; 1:250) and mounted with the ProLong Gold Antifade Reagent with DAPI (Cell Signaling Technologies, 8961). Cells were imaged using an Olympus FlowView1000 confocal microscope and FlowView ASW V03.01.03.03 or V4.2a software at the UCSD Microscopy Core.

Molecular Data Generation and Processing

RNA-seq

Total RNA was isolated using the Quick-RNA Mini Prep Kit (Zymo) from frozen RTL plus pellets, including on-column DNAse treatment step. RNA was eluted in 40 µl RNAse-free water and run on a Bioanalyzer (Agilent) to determine integrity. Concentration was measured by Qubit. Illumina Truseq Stranded mRNA libraries were prepared and sequenced on HiSeq4000, to an average of 40 M 150 bp paired-end reads per sample. RNA-seq reads were aligned using STAR (Dobin et al., 2013) with a splice junction database built from the Gencode v19 gene annotation (Harrow et al., 2012b). Transcript and gene-based expression values were quantified using the RSEM package (1.2.20) (Li and Dewey, 2011) and normalized to transcript per million bp (TPM). Public data sets were also processed through this pipeline prior to analysis.

ATAC-seq

We performed ATAC-seq using the protocol from Buenrostro et al. (Buenrostro et al., 2013) with small modifications. Frozen nuclear pellets of 5×10^4 cells each were thawed on ice and tagmented in permeabilization buffer containing digitonin. Tagmentation was carried in 25µl using 2.5µl of Tn5 from Nextera DNA Library Preparation Kit (Illumina) for 60 min at 37°C in a thermomixer (500 RPM shaking). To eliminate confounding effects due to index hopping, all libraries within a pool were indexed with unique i7 and i5 barcodes. Libraries for iPSC-RPE were amplified for 12 cycles. Libraries were sequenced to approximately 80M 150bp paired end reads on the HiSeq 4000 (Illumina) platform. ATAC-seq reads were aligned using STAR to hg19. Duplicate reads, reads mapping to blacklisted regions from ENCODE, reads mapping in chromosome other than chr1-chr22, chrX, chrY, and read-pairs with mapping quality Q<30 were filtered. In addition, to restrict the analysis to regions spanning only one nucleosome, we required an insert size no larger than 140 bp, as we observed that this filtering improved sensitivity to call peaks and reduced noise. Peak calling was performed using MACS2 on BAM files with the command 'macs2 callpeak --nomodel --nolambda --keep-dup all --call-summits -f BAMPE -g hs', and peaks were filtered by enrichment score (q < 0.01). Samples were required to have a fraction of reads within peaks greater than 2% (range observed: 6.6-23%). Public data sets were also processed through this pipeline prior to analysis.

ChIP-Seq

For H3K27ac, 5-15 x 10^6 formaldehyde crosslinked iPSC-RPE cells were lysed and sonicated in 110 µl of SDS Lysis Buffer (0.5% SDS, 50mM Tris-HCl pH 8.0, 20mM EDTA, 1x cOmplete™ Protease Inhibitor Cocktail (Sigma)) using Covaris E220 Focused-ultrasonicators (Covaris) for 14 cycles, 1 min per cycle, duty cycle 5. For each sample, H3K27ac antibody (Abcam ab4729, lot GR00324078) was coupled for 4 hours to 20µl of Protein G Dynabeads (Thermo Scientific) and used for overnight chromatin immunoprecipitation in IP buffer (1% Triton X-100, 0.1% DOC, 1x TE buffer, 1x cOmplete™ Protease Inhibitor Cocktail). Beads with immunoprecipitated chromatin were washed with six times with 150 µl of ChIP wash buffer (50mM HEPES pH 8.0, 1% NP-40, 0.7% DOC, 400mM LiCl, 1mM EDTA, 1x cOmplete™ Protease Inhibitor Cocktail) and twice with 1X TE buffer (10mM Tris-HCl pH 8.0, 1mM EDTA). Next, samples were eluted in 150 µl of ChIP Elution Buffer (1% SDS, 10mM Tris-HCl pH 8.0, 1mM EDTA) and reverse crosslinked by incubation for 15 min at 65˚C with rotation and subsequent incubation with 5 µl RNAse (Sigma) for 1h at 37˚C and Proteinase K Solution (20 mg/mL, Thermo Fisher Scientific) for 1h at 55˚C. After reverse crosslinking samples were purified with 2X Agencourt AMPure XP DNA beads (Beckman Coulter), eluted in 30 µl of H_2O and Qubit (Thermo Scientific) quantified. Libraries were generated using KAPA Hyper Prep Kit (KAPA Biosystems) and KAPA Real Time Library Amplification Kit (KAPA Biosystems) following manufacturers manual. Libraries were barcoded using TruSeq RNA Indexes (Illumina). Libraries were sequenced on an Illumina HiSeq 4000 to an average of 40 M 150 bp Paired-End reads per sample. ChIP-Seq reads were mapped to the hg19 reference using BWA. Duplicate reads, reads mapping to blacklisted regions from ENCODE, reads not mapping to chromosomes chr1-chr22, chrX, chrY, and read-pairs with mapping quality Q <30 were filtered. Peak calling was performed for each sample using MACS2(Zhang et al., 2008) ('macs2 callpeak -f BAMPE -g hs -B --SPMR --verbose 3 --cutoff-analysis --call-summits -q 0.01') with reads derived from sonicated chromatin not subjected to IP (ie input chromatin) from a pool of samples used as a negative control.

Data analysis

<u>RNA-seq analysis</u>

Principal component analysis (PCA) was performed on the 10,000 genes with the most variable expression (i.e. with the highest standard deviation across samples) across all analyzed samples (including or excluding iPSC and iPSC-CM samples). Functional enrichment of genes associated with each PC was performed using goseq (Young et al., 2010) by comparing the 1,000 genes with the highest or lowest loading on the PC to the other 9,000 used for PCA. To generate t-SNE plots, Rtsne (Krijthe, 2015) was used to perform t-SNE on the first 30 components from the PCA analysis. RPE signature genes were obtained from (Strunnikova et al., 2010) and annotated according to (Liao et al., 2010). For each iPSC-RPE, we tested the correlation between the expression of the log2(TPM) values for the iPSC-RPE and the Fetal RPE sample for each set of signature genes using cor.test in R. All correlations were above 0.83 (range 0.83 – 0.97, mean 0.90) and had P-values below 1×10^{-6}.

ATAC-seq analysis

ATAC-seq data was obtained from Wang et al. via GEO (GSE99287) and SRA (SRP107997). Sequencing reads were processed through the same computational pipeline as data generated for this study. The five healthy donors were treated as unique subjects. For the AMD samples, as samples from the same subject could be annotated as early or late depending on the affected status of the eye, the five AMD donors were treated as unique subjects, whether or not the sample was annotated as early or late AMD.

TF enrichment

Enrichment for transcription factor motifs was performed with HOMER (Heinz et al., 2010) using HOCOMOCOv11 motifs at P = 0.0001 (http://hocomoco11.autosome.ru/final_bundle/hocomoco11/core/HUMAN/mono/HOCOMOCOv11_core_HUMAN_mono_homer_format_0.0001.motif). Sequences flanking the summits identified by MACS2 were examined using findMotifsGenome.pl –size 200 –p 1 –mask. To cluster TF motif profiles, the enrichment P-values were ranked within each sample across all TFs clustered and clustered using hierarchical clustering with the R package pheatmap (Kolde, 2015).

Measuring AMD GWAS enrichment within tissue ATAC-seq peaks

To measure the enrichment of AMD GWAS within different tissues, we applied fgwas (Pickrell, 2014) on each set of ATAC-seq peaks independently. Variants were used if their alternate allele frequency could be obtained from the 1000 Genomes Project Phase 3 EUR data by matching the chromosome, position, and annotated alleles. This resulted in a loss of 4% of variants tested. Annotations for exonic, promoter, and UTR regions were obtained from GENCODE (Harrow et al., 2012a) annotations (V19). Annotations for missense and synonymous variants were obtained from the 1000 Genomes Project Phase 3 (Genomes Project et al., 2015) functional annotation. ATAC-seq peak regions or H3K27ac peak regions were called separately for each sample and then merged using bedtools v1.7 (Quinlan and Hall, 2010). To identify variants within each set of ATAC-seq peaks or other annotations for input to fgwas, we used bedtools v1.7 (Quinlan and Hall, 2010). For input to fgwas, we calculated Z scores using the β and its standard error, and ran fgwas using these Z scores and standard errors on consecutive ~1 Mb intervals across the genome (-k 3355).

Fine-mapping AMD GWAS loci

To perform fine-mapping of the AMD GWAS loci, we trained an fgwas model containing annotations from ATAC-seq data (Early AMD RPE, Early AMD Retina, Late AMD RPE, Late AMD Retina, Healthy RPE, Healthy Retina, human fetal RPE, and iPSC-RPE), H3K27ac data (human fetal RPE and iPSC-RPE), and genome annotations (exons, promoters, untranslated 3' or 5' regions, missense variants, or synonymous variants). We applied fgwas on these annotations, and chose the cross-validation penalty to use by first trying penalties between 0.05 and 0.30 in steps of 0.05, and then positive non-zero penalties in 0.01 steps surrounding the best penalty by 0.05, resulting in a tested range of penalties from 0.25-0.30; a final penalty of 0.30 was selected as it had the best likelihood. Finally, we removed annotations from the model until the likelihood stopped increasing, resulting in 11 annotations from the three types being retained: ATAC-seq (Early AMD RPE, Early AMD Retina, Late AMD RPE, Late AMD Retina, Healthy Retina, human fetal RPE, and iPSC-RPE), human fetal H3K27ac, and genome annotations (exons, missense

variants, and promoters). We used the model with fgwas to update the Bayes Factors for each variant using the cross-validation estimated ridge parameter and calculated the posterior probability of causality (PP) for each variant within 1 Mb windows flanking the reported lead variant (Fritsche et al., 2016). For two of the lead variants, we were unable to obtain allele frequencies from the 1000 Genomes Project, resulting in them being removed from the analysis (TRPM3/rs71507014 and MMP9/rs142450006). We therefore did not perform fine-mapping for these loci. The PPA is the proportion of the total GWAS risk signal at a locus measured by Bayes Factors that is attributed to a particular variant, multiplied by the probability that the genomic region contained a real signal (set to 1 for GWAS loci). Variants that were associated with annotation information were classified into functional classes based on the following criteria in the following order: Coding = missense variant, Local Regulatory = promoter annotation at least one ATAC-seq or H3K27ac annotation, Distal Regulatory = at least one ATAC-seq or H3K27ac ChIP-seq annotation, Unknown = all others.

VEGFA locus annotation

To visualize the *VEGFA* region (Figure 4A), -log10 P-values from (Fritsche et al., 2016) were plotted along with the PPA of all SNPs after prioritization with fgwas. TPM normalized RNA-seq expression data from an iPSC-RPE (iPSCORE_1_14) was used to identify expressed genes in the region. Hi-C loops were obtained from Greenwald et al. (Greenwald et al., in press).

ATAC-seq peak ASE of rs943080

To examine ASE at the ATAC-seq peak containing rs943080, allelic read depth was measured using samtools (Li, 2011) mpileup. Read depth from all samples associated with the same subject were combined. For the iPSC-RPE, the genotype at rs943080 was obtained from whole genome sequence data (dbGaP phs001325). For each subject, an ASE P-value was calculated by testing the read depth counts to the expected frequency (50%) using a binomial test (binom.test in R). Across all heterozygotes, a meta-analysis P was calculated using the "sumlog" method in the R package metap (Dewey, 2018). For adult healthy subjects, only the donors with at least 2 reads from each allele

were considered heterozygotes and the meta-analysis P was calculated in the same manner as the iPSC-RPE. For the adult early and late-stage AMD samples, since we did not observe any samples with alternate reads, we calculated the probability of observing four subjects with a homozygous reference genotype as the square of the reference allele frequency in the 1000 Genomes Project (Genomes Project et al., 2015) EUR population (0.48) to the fourth power. To determine if rs943080 was present in the Roadmap tissues, we examined the SNP location in Haploreg (v4.1) (Ward and Kellis, 2016).

RNA-seq analysis at *VEGFA*

Expression differences between the iPSC-RPE from the five rs943080 heterozygous subjects were compared to the one homozygous subject using edgeR (Robinson et al., 2010). Reads counts were merged across all transcripts to examine gene-level differences. The unadjusted quasi-likelihood F-test P-value is reported, although the likelihood ratio test result was similar. ASE analysis of RNA-Seq data was performed using MBASED (Mayba et al., 2014). Heterozygous SNVs were identified by intersecting variant calls from WGS with exonic regions from Gencode v19. The WASP pipeline (van de Geijn et al., 2015) was employed to reduce reference allele bias at heterozygous sites. The number of read pairs supporting each allele was counted using the ASEReadCounter from GATK (3.4-46) (DePristo et al., 2011). Heterozygous SNVs were then filtered to keep SNVs where the reference or alternate allele had more than 8 supporting read pairs, the reference allele frequency was between 2-98%, and the SNV was located in unique mappability regions according to wgEncodeCrgMapabilityAlign100mer track, and not located within 10 bp of another variant in a particular subject (heterozygous or homozygous alternative) (Consortium, 2015; Lappalainen et al., 2013; Mayba et al., 2014). The raw gene-based P-values were reported. For visualization, only SNPs with a combined read depth of more than 30 were shown.

Buenrostro, J.D., Giresi, P.G., Zaba, L.C., Chang, H.Y., and Greenleaf, W.J. (2013). Transposition of native chromatin for fast and sensitive epigenomic profiling of open chromatin, DNA-binding proteins and nucleosome position. Nat Methods *10*, 1213-1218.
Consortium, G.T. (2015). Human genomics. The Genotype-Tissue Expression (GTEx) pilot analysis: multitissue gene regulation in humans. Science *348*, 648-660.

DePristo, M.A., Banks, E., Poplin, R., Garimella, K.V., Maguire, J.R., Hartl, C., Philippakis, A.A., del Angel, G., Rivas, M.A., Hanna, M., *et al.* (2011). A framework for variation discovery and genotyping using next-generation DNA sequencing data. Nat Genet *43*, 491-498.
Dewey, M. (2018). metap: meta-analysis of significance values.
Dobin, A., Davis, C.A., Schlesinger, F., Drenkow, J., Zaleski, C., Jha, S., Batut, P., Chaisson, M., and Gingeras, T.R. (2013). STAR: ultrafast universal RNA-seq aligner. Bioinformatics *29*, 15-21.
Fritsche, L.G., Igl, W., Bailey, J.N., Grassmann, F., Sengupta, S., Bragg-Gresham, J.L., Burdon, K.P., Hebbring, S.J., Wen, C., Gorski, M., *et al.* (2016). A large genome-wide association study of age-related macular degeneration highlights contributions of rare and common variants. Nat Genet *48*, 134-143.
Genomes Project, C., Auton, A., Brooks, L.D., Durbin, R.M., Garrison, E.P., Kang, H.M., Korbel, J.O., Marchini, J.L., McCarthy, S., McVean, G.A., *et al.* (2015). A global reference for human genetic variation. Nature *526*, 68-74.
Greenwald, W.W., Li, H., Benaglio, P., Jakubosky, D., Matsui, H., Schmitt, A., Selvaraj, S., D'Antonio, M., D'Antonio-Chronowska, A., Smith, E.N., *et al.* (in press). Integration of phased Hi-C and molecular phenotype data to study genetic and epigenetic effects on chromatin looping. Nature Communications.
Harrow, J., Frankish, A., Gonzalez, J.M., Tapanari, E., Diekhans, M., Kokocinski, F., Aken, B.L., Barrell, D., Zadissa, A., Searle, S., *et al.* (2012a). GENCODE: the reference human genome annotation for The ENCODE Project. Genome Res *22*, 1760-1774.
Harrow, J., Frankish, A., Gonzalez, J.M., Tapanari, E., Diekhans, M., Kokocinski, F., Aken, B.L., Barrell, D., Zadissa, A., Searle, S., *et al.* (2012b). GENCODE: the reference human genome annotation for The ENCODE Project. Genome Res *22*, 1760-1774.
Heinz, S., Benner, C., Spann, N., Bertolino, E., Lin, Y.C., Laslo, P., Cheng, J.X., Murre, C., Singh, H., and Glass, C.K. (2010). Simple combinations of lineage-determining transcription factors prime cis-regulatory elements required for macrophage and B cell identities. Mol Cell *38*, 576-589.
Kolde, R. (2015). pheatmap: Pretty Heatmaps.
Krijthe, J.H. (2015). Rtsne: T-Distributed Stochastic Neighbor Embedding using a Barnes-Hut Implementation.
Lappalainen, T., Sammeth, M., Friedlander, M.R., t Hoen, P.A., Monlong, J., Rivas, M.A., Gonzalez-Porta, M., Kurbatova, N., Griebel, T., Ferreira, P.G., *et al.* (2013). Transcriptome and genome sequencing uncovers functional variation in humans. Nature *501*, 506-511.
Li, B., and Dewey, C.N. (2011). RSEM: accurate transcript quantification from RNA-Seq data with or without a reference genome. BMC Bioinformatics *12*, 323.
Li, H. (2011). A statistical framework for SNP calling, mutation discovery, association mapping and population genetical parameter estimation from sequencing data. Bioinformatics *27*, 2987-2993.
Liao, J.L., Yu, J., Huang, K., Hu, J., Diemer, T., Ma, Z., Dvash, T., Yang, X.J., Travis, G.H., Williams, D.S., *et al.* (2010). Molecular signature of primary retinal pigment epithelium and stem-cell-derived RPE cells. Hum Mol Genet *19*, 4229-4238.
Maruotti, J., Sripathi, S.R., Bharti, K., Fuller, J., Wahlin, K.J., Ranganathan, V., Sluch, V.M., Berlinicke, C.A., Davis, J., Kim, C., *et al.* (2015). Small-molecule-directed, efficient generation of retinal pigment epithelium from human pluripotent stem cells. Proc Natl Acad Sci U S A *112*, 10950-10955.
Mayba, O., Gilbert, H.N., Liu, J., Haverty, P.M., Jhunjhunwala, S., Jiang, Z., Watanabe, C., and Zhang, Z. (2014). MBASED: allele-specific expression detection in cancer tissues and cell lines. Genome Biol *15*, 405.
Panopoulos, A.D., D'Antonio, M., Benaglio, P., Williams, R., Hashem, S.I., Schuldt, B.M., DeBoever, C., Arias, A.D., Garcia, M., Nelson, B.C., *et al.* (2017). iPSCORE: A Resource of 222 iPSC Lines Enabling Functional Characterization of Genetic Variation across a Variety of Cell Types. Stem cell reports *8*, 1086-1100.
Pickrell, J.K. (2014). Joint analysis of functional genomic data and genome-wide association studies of 18 human traits. Am J Hum Genet *94*, 559-573.
Quinlan, A.R., and Hall, I.M. (2010). BEDTools: a flexible suite of utilities for comparing genomic features. Bioinformatics *26*, 841-842.
Robinson, M.D., McCarthy, D.J., and Smyth, G.K. (2010). edgeR: a Bioconductor package for differential expression analysis of digital gene expression data. Bioinformatics *26*, 139-140.
Strunnikova, N.V., Maminishkis, A., Barb, J.J., Wang, F., Zhi, C., Sergeev, Y., Chen, W., Edwards, A.O., Stambolian, D., Abecasis, G., *et al.* (2010). Transcriptome analysis and molecular signature of human retinal pigment epithelium. Hum Mol Genet *19*, 2468-2486.
van de Geijn, B., McVicker, G., Gilad, Y., and Pritchard, J.K. (2015). WASP: allele-specific software for robust molecular quantitative trait locus discovery. Nat Methods *12*, 1061-1063.
Ward, L.D., and Kellis, M. (2016). HaploReg v4: systematic mining of putative causal variants, cell types, regulators and target genes for human complex traits and disease. Nucleic Acids Res *44*, D877-881.

Young, M.D., Wakefield, M.J., Smyth, G.K., and Oshlack, A. (2010). Gene ontology analysis for RNA-seq: accounting for selection bias. Genome Biol *11*, R14.
Zhang, Y., Liu, T., Meyer, C.A., Eeckhoute, J., Johnson, D.S., Bernstein, B.E., Nusbaum, C., Myers, R.M., Brown, M., Li, W., *et al.* (2008). Model-based analysis of ChIP-Seq (MACS). Genome Biol *9*, R137.
